# Netrin-4 promotes mural cell adhesion and recruitment to endothelial cells

**DOI:** 10.1186/2045-824X-6-1

**Published:** 2014-01-28

**Authors:** Esma Lejmi, Ilyes Bouras, Serge Camelo, Marie Roumieux, Norbert Minet, Carole Leré-Déan, Tatyana Merkulova-Rainon, Gwennhael Autret, Catherine Vayssettes, Olivier Clement, Jean Plouët, Laurence Leconte

**Affiliations:** 1 Present address: SISENE, Pépinière Paris Santé Cochin, 29 rue du Faubourg Saint Jacques, Paris 75014, France; 2 INSERM U965/Institut des Vaisseaux et du Sang, Hôpital Lariboisière, 8 rue Guy Patin, Paris cedex 10 75475, France; 3 Plate-forme d’imagerie du petit animal, PARCC - Paris - Centre de recherche Cardiovasculaire à l’HEGP, Inserm-UMR 970, 56 rue Leblanc, 75015, Paris, France

**Keywords:** Netrin-4, Blood vessel, Mural cell, Angiogenesis, Basement membrane, Tumor growth

## Abstract

Netrins are secreted molecules involved in axon guidance and angiogenesis. We previously showed that Netrin-4 acts as an anti-angiogenic factor by inhibiting endothelial cell (EC) functions. In this study, we investigated the effects of Netrin-4 on vascular smooth muscle cell (VSMC) activity *in vitro* and *in vivo*. We show that exogenous Netrin-4 stimulated VSMC adhesion and migration, and increased their coverage on EC tubes (grown on a Matrigel substrate). siRNA knock-down of endogenous Netrin-4 expression in VSMC decreased their recruitment to EC tubes. VSMC expressed Netrin-4 and three of the six Netrin-1 cognate receptors: DCC, Neogenin, and Unc5B. Silencing of these receptors reduced Netrin-4 adhesion to VSMC, strongly suggesting that these receptors were involved in the recruitment process. We previously showed that Netrin-4 overexpression in PC3 cancer cells delayed tumor growth in a model of subcutaneous xenograft by reducing tumor vessel density. Here, we show that Netrin-4 overexpression improved tumor blood vessel structure and increased VSMC coverage. Thus, Netrin-4 induced mural cell recruitment may play a role in the inhibition of tumor growth. Our data suggest that Netrin-4 is important for blood vessel normalization through the regulation of both endothelial and perivascular cells.

## Introduction

The integrity of the blood vessel network requires reciprocal interactions between endothelial cells (EC) and associated perivascular cells (referred to as pericytes, vascular smooth muscle cells (VSMC) or mural cells). EC form the inner lining of the vessel wall and perivascular cells envelop the surface of the vascular tube [1]. During angiogenesis, newly formed blood vessels become stabilized through the recruitment of vascular mural cells. Interactions between EC and mural cells in the blood vessel may be important in the regulation of vascular formation, stabilization, remodeling and function. Failure of these interactions during development results in severe or lethal vascular defects. Diseases such as diabetes and tumor angiogenesis result from abnormal interactions between the two cell types [2,3]. The role of EC in these interactions has been extensively studied, whereas the importance of pericyte coverage in blood vessel maturation has only been demonstrated more recently [1-4]. Pericyte coverage may limit tumor cell metastasis [5,6]. Therefore, it is possible that factors inhibiting angiogenesis not only reduce EC functions, but also stimulate pericyte coverage.

Originally identified as axonal guidance molecules, Netrins are laminin-like secreted proteins involved in angiogenesis and blood vessel network formation [7-10]. The Netrin system comprises of at least five ligands (Netrin 1, 2, 4, G1a, G1b) and six receptors (Neogenin, DCC, Unc5A, B, C and D) [11]. As observed in nervous system axonal guidance, Netrins act as bi-functional cues for angiogenesis; however, the role of Netrins within the vasculature remains unclear. Previous studies have focused on the role of Netrin-1 and Netrin-4 in angiogenesis, with conflicting results. It has been reported that Netrins stimulate cell proliferation and migration in primary EC cultures [12,13] and in VSMC [14]. These pro-angiogenic effects have been shown to be independent of cognate Netrin receptor expression on EC [12,13] with Neogenin as the only receptor involved in VSMC Netrin signaling [14]. However, Netrins also act as anti-angiogenic factors modulating the response of EC to VEGF [15-17]. Netrin-4 is anti-angiogenic, inhibiting EC functions through binding to Neogenin and recruitment of Unc5B [17]. Netrin-4 can also cause filopodial retraction of EC *in vivo*[15]. Netrin-1 has also been shown to inhibit postnatal sprouting angiogenesis and neovascularization via the activation of the Unc5B receptor [16]. In addition, overexpression of Netrin-1 or Netrin-4 by tumor cells delays tumor angiogenesis in various animal models [16-19].

Netrin-4 is expressed in all ocular tissues and contributes to angiogenesis in the retina [20,21]. In particular, it is a prominent protein of vascular basement membranes where it appears to be tightly associated with pericytes. Characterization of null mice retinal phenotypes demonstrated a role for Netrin-4 as a negative regulator of vascular branching [20]. Little is known about the role of Netrins on VSMC, EC–pericyte interactions and the formation of the basement membrane (BM).

The composition and assembly of the vascular basal lamina plays a key role in vascular tube maturation and stabilization [22]. Both EC and pericytes induce the expression of basement membrane components, thus contributing to extracellular matrix synthesis, deposition and remodeling. Netrin-4 localizes to basement membranes [23] and to the vascular basal lamina, where it interacts with components of the extracellular matrix such as laminins and integrins [23-25]. Netrin-4 is secreted by both EC [17] and VSMC (this study). EC–pericyte interactions may be important for the assembly of the basement membrane and for the biological functioning of blood vessels. There is evidence that a deficit in vascular basal lamina components influences tumorigenesis [26,27]. Basement membrane abnormalities and lack of pericyte coverage are also frequently observed in the retina of patients with diabetic retinopathy [28]. To clarify the mechanisms by which Netrin-4 inhibits angiogenesis and reduces tumor growth, we examined the effects of Netrin-4 on VSMC *in vitro* and on tumor angiogenesis.

## Materials and methods

### Cell culture

VSMC were isolated from human umbilical cord blood as previously described [29]. Briefly, mononuclear cells were obtained from umbilical blood by Ficoll separation. Cells were allowed to pre-adhere overnight on a culture dish and then fibroblasts were discarded. The remaining adherent cells were re-suspended, washed and cultured in M199 medium, supplemented with 20% FCS and 10 ng/ml VEGF in 6-well plates coated with rat tail collagen I. The medium was changed twice a week. After 3–4 weeks, the VSMC clones were collected and expanded. VSMC identity was confirmed by alpha smooth muscle actin immunostaining [29]. Human Umbilical Arterial Endothelial Cells **(**HUAEC) from the same donors were isolated by collagenase dissociation (Roche Diagnostic). EC were cultured in EBM (Clonetics) supplemented with 10% FCS and 2 ng/ml of VEGF. The medium was changed every 4 days. Their EC origin was confirmed by von Willebrand Factor (vWF) staining. Primary Porcine Retinal Pericyte cells (PRPC) were cultured in DMEM supplemented with 15% FCS.

### Wound migration assays

The IncuCyte live-cell imaging system was used for cell migration assays (Essen BioScience). Cells were grown to confluence in 96-well or 24-well Essen Bioscience plates previously coated with 0.2% Gelatin (Sigma). The culture plates were loaded into the wound maker tool, which creates precise and reproducible wounds in all wells. Plates were washed twice with culture medium and then incubated in medium with or without human recombinant Netrin-4 (R&D; 50 ng/ml. Each plate was then placed inside the IncuCyte and kinetic images were taken every 2 hours for 24 h. IncuCyte software was used to quantify cell migration.

### Proliferation cell assay

The number of living cells was spectrophotometrically measured using an MTT assay. Cells were seeded onto culture plates previously coated with 0.2% gelatin and grown in their regular medium. The next day, cells were stimulated with the indicated concentrations of Netrin-4 (recombinant human Netrin-4 from R&D). Twenty four hours later, cells were washed once with PBS and incubated at 37°C in 5% CO_2_ in a solution of MTT (Sigma; 1 mg/ml in PBS). After 2 hours, isopropanol (50%) was directly added to the MTT solution and plates were gently mixed using a plate shaker. The absorbance was directly measured at 570 nm in a microplate reader. Data were analysed using Excel software.

### Cell adhesion assay

Cell adhesion assays were performed as previously described [30]. The wells of 96-well Maxisorp plates (Nunc) were coated overnight at 37°C with either 1% BSA (Bovine serum Albumin, Sigma), 2.5 μg/ml human Vitronectin (R&D) or recombinant human Netrin-4 (R&D) diluted in PBS. After two washes in PBS, non-specific binding sites were blocked for 1 hour at 37°C using 1% BSA. After washes with PBS and water, 100 μl of a cell suspension containing 500000 cells per ml in culture medium was added (three wells per treatment) to each well and incubated at 37°C for 4 hours. Non-adherent cells were washed off with water. Cells that adhered to the substrate were fixed and stained with crystal violet (0.2% in methanol). Images were acquired with an inverted microscope (Nikon Eclipse Ti) equipped with a digital camera. Dye bound to adhered cells was solubilized with 0.1% SDS and the absorbance at 560 nm was measured. The data reported were mean values of the three determinations per treatment.

### HUAEC and VSMC co-cultures on Matrigel

The *in vitro* angiogenesis assay was performed according to a previously published protocol [31].

Briefly, 24-well cell culture plates were coated with Matrigel Basement Membrane Matrix (BD Biosciences, Le Pont de Claix France). The culture plates were incubated at 37°C for at least 30 minutes to allow the basement membrane to form a gel. HUAEC were labeled with SP-Dioc_18_ (3,3’-dioctadecyl-5-5’-di(4-sulfophenyl) oxacarbocyanine, Invitrogen) green dye (2 μg/ml) and VSMC were labeled with CM-Dil red dye (1 μg/ml) (Invitrogen). HUAEC were added on top of the Matrigel matrix (3.10^4^ per well) and then incubated overnight in EBM 10% FCS to induce tube formation. VSMC were then added to the endothelial network (1.5×10^4^ VSMC per well) and the samples incubated for 5 hours. Cells were visualized by inverted-phase fluorescence microscopy (Zeiss, Le Pecq, France). Photographs of ten representative fields were taken and quantified using Histolab software (Microvision, Evry). Statistical analyses were performed using either Student’s t test or ANOVA.

### Small interfering RNA and transfection assays

Subconfluent cells were transfected with a mixture of 3 μl/ml *Trans*IT-KO transfection reagent (Mirus) and 50 nM siRNA diluted in serum free culture medium, according to the manufacturer’s protocol. siRNA targeting different sequences in the human *netrin-4* gene were obtained from Qiagen (NTN 1–4). Two sets of 4 predesigned siRNA (*SMART*pool, Dharmacon) were used to silence *DCC* and *Unc5b* gene expression. Two silencer validated neogenin siRNAs were purchased from Ambion (Autin, TX). Different siRNAs were used as controls: the siCONTROL non-targeting siRNA from Dharmacon and the Stealth RNAi negative control from Invitrogen.

### RT-PCR analysis

Total cellular RNA was isolated using the RNeasy mini kit (Qiagen) following the manufacturer's instructions. The first strand cDNA template was synthesized from 0.5 μg of total RNA using the Superscript II Reverse Transcriptase synthesis kit and random hexamer primers (Invitrogen). The cDNA product was amplified using the Taq DNA polymerase mix (Invitrogen) and a Px2 thermal cycler apparatus. The primers used for PCR are given in Table 1. Amplified products were analyzed on a 2% agarose gel with ethidium bromide staining. The mRNA levels were normalized to beta Actin mRNA.

**Table 1:** Primers used for RT-PCR and qPCR

**Name**	**Accession number**	**Forward primer**	**Reverse primer**
Neogenin	62U612	5'-TCGCTGCGTAGTGGAAAGTG-3'	5'-CTGTGTCAAGTGCCTCCTCA-3'
DCC	X76132	5'-ACTTGGGGTGGTGAAGTCAG-3'	5'-CCAAGACAGGGACCACATCT-3'
Unc5A	AK131380	5'-CATCGAGTGCTTTGAGGTGA-3'	5'-ACCTGCTGCCTTGAGACATT-3'
Unc5B	AY126437	5'-ACTCATCTGCTGCCCTGACT-3'	5'-ATTTTGTCGGTGGAGTCCTG-3'
Unc5C	AF055634	5'-CACTGCACCTTCACTCTGGA-3'	5'-GAGAGGGATGCTGAAAGCAC-3'
Unc5D	AB055056	5'-GGTGCTCCTGAGTCCTGAAG-3'	5'-GGGTCCAAAAGGCAGTAACA-3'
Netrin 4	AF278532	5'-CAGACATAGACTGGTGTCATGAAGTT-3'	5'-ACATTGACCTCAACATGAGTACCTT-3'
Beta actin	X00351	5'-AGAGCTACGAGCTGCCTGAC-3'	5'-AGCACTGTGTTGGCGTACAG-3'

### Quantitative reverse transcription PCR

The cDNA product was amplified using Power SYBR Green PCR Master Mix (Applied Biosystems, France) and a Light Cycler 1.5 apparatus with the primers described in Table 1. Standard curves for each mRNA were generated by serial dilutions of cDNA synthesized from total RNA isolated from human fetal brain tissue.

### Transfection of PC3 prostate carcinoma cells and xenograft in nude mice

Transfection of PC3 cells and mouse xenografts was performed as previously reported [17]. Briefly, different clones of PC3 cells overexpressing Netrin-4 were established and characterized. Female, athymic, nude mice were randomized (eight per group) and injected subcutaneously with 2 × 10^6^ PC3 cells transfected with either Netrin-4 or empty vector pcDNA3.

### Intravital microscopy

Intravital microscopy was performed with a Cellvizio® (Mauna Kea Technologies) Leica FCM1000. This device combines confocal fluorescence microscopy with an optical-fiber miniprobe (1.5 mm diameter, 488 nm excitation wavelength), which collects light (505–700 nm wavelength) from the observation site and conveys it to a dedicated imaging device. Cellvizio® can explore microvascularization of denuded tumors in their native environment with limited invasiveness and preservation of the physiological state of tissues. Mice were anesthetized with 75 mL/kg of a solution of 5% xylazine (Rompum 2%) and 20% ketamine (Imalgene 500). Animal body temperature was maintained using a heating plate. Fluorescein isothiocyanate-dextran 500 kDa (FD-500S, Sigma Aldrich®, 35 mg/kg) was injected intravenously (retro-orbitary injection) to visualize blood vessels. The excitation maximum of FITC-dextran is 490 nm and the emission maximum is 520 nm. Videos and images of tumor angiogenesis were obtained using the Cellvizio® software, ImageCell™.

### Immunohistochemistry

Netrin-4 in VSMC was detected after fixation in 4% PFA followed by addition of 0.1% Triton. An antibody against human Netrin-4 (R&D) was detected with Alexa Fluor-labelled anti-goat secondary antibody (Molecular Probes, 2 μg/μl). Cells were observed using an inverted fluorescence microscope (Nikon Eclipse Ti) equipped with a digital camera.

Intratumoral vessels in 10 μm-thick cryostat sections were detected using rabbit anti-desmin (labvision, 1/200 in PBS 1% triton) and rat anti-CD31 (BD Biosciences) as primary antibodies. Alexa Fluor 555-labeled donkey anti-rabbit and Alexa Fluor 488 chicken anti-rat (1:250 dilution, Invitrogen, Molecular Probes, Eugene, USA) were then applied as secondary antibodies. Images were acquired with an inverted fluorescence microscope (Zeiss, Le Pecq, France). Desmin staining was quantified at a 20x magnification using Image J software. The Mann–Whitney U test was used for statistical analyses.

### Statistical analysis

Microsoft Excel was used for data analysis and graphic representation. Data were presented as means ± standard deviation (SD). ANOVA was used for statistical analyses. *P* values less than 0.05 were considered statistically significant. *P < 0.05, **P < 0.01, ***P < 0.001.

## Results

### Netrin-4 promotes mural cells migration

**Figure 1:**
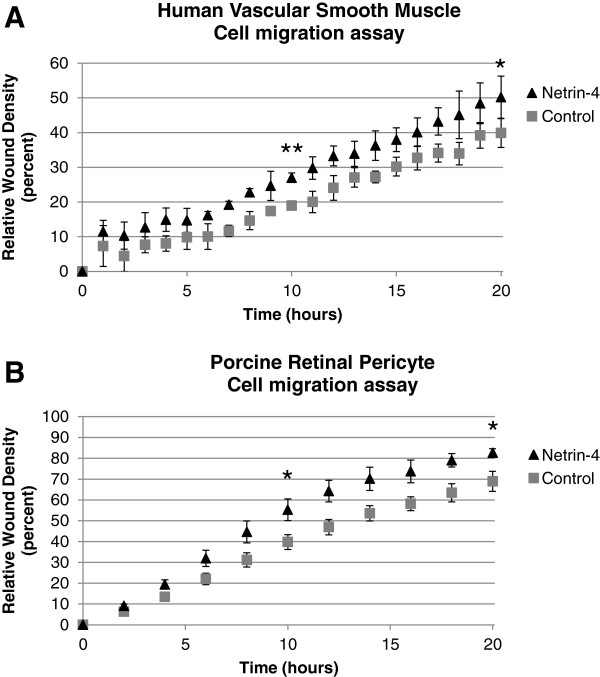
**Netrin-4 promotes Mural cell migration.** Time course of Vascular Smooth Muscle Cell **(A)** and Porcine Retinal Pericyte Cells **(B)** migration with and without the addition of Netrin-4 (R&D; 50 ng/ml). Cells were grown to confluence in complete medium and wounds were made. Cells were treated (N4) or not (Control) with the human recombinant protein Netrin-4 (50 ng/ml) and allowed to migrate for 20 hours. Data were collected and analyzed on IncuCyte. Compared to controls, treatment with Netrin-4 stimulates VSMC **(A)** and PRPC **(B)** migration. Statistical analysis of data at t = 10 h and t = 20 h showed that Netrin-4 significantly increased VSMC **(A)** and PRPC **(B)** migration.

We investigated the effect of Netrin-4 on the migration of mural cells using two independent cell types. First, a wound-healing assay was performed using primary Vascular Smooth Muscle Cells (VSMC) isolated from human umbilical cord blood. Migration of VSMC was analyzed using an automated wound-healing assay (IncuCyte technology; Essen BioScience) during a 20-hour period in which the cells were incubated in the presence or absence of human recombinant Netrin-4. The addition of Netrin-4 stimulated VSMC migration (Figure 1A). Second, the effect of Netrin-4 on the migration of porcine retinal pericyte cells (PRPC) was measured using the same experimental system. The presence of 50 ng/ml of Netrin-4 increased the migration of PRPC (Figure 1B). In both cell types, the addition of Netrin-4 stimulated migration; however, the effect of cell proliferation was unknown. A MTT cell proliferation assay indicated that addition of Netrin-4 did not increase cell proliferation after 24 hours (Additional file 1: Figure S1).

### Netrin-4 promotes VSMC adhesion

**Figure 2:**
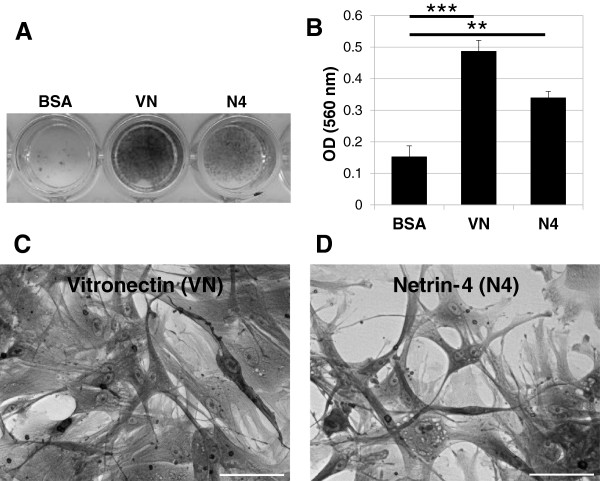
**Adhesion of cultured VSMC to Netrin-4. (A)** Crystal violet staining of cells that adhered to the substrate. 96-well plates were coated overnight with BSA, Vitronectin (VN; 2.5 μg/ml) or Netrin-4 (N4, 1 μg/ml). VSMC were added to the wells and incubated for 4 hours at 37°C. BSA was used as a negative control and Vitronectin (VN) was used as a positive control. VSMC adhere to Netrin-4 (N4) or Vitronectin but not to BSA. **(B)** Quantification of cell adhesion with the three substrates by measurement of absorbance. All assays were performed in triplicate in at least three different experiments. **(C)** Contrast phase image of VSMC adhesion in the presence of Vitronectin, 20x magnification **(D)** Contrast phase image of VSMC adhesion in the presence of Netrin-4, 20x magnification. Scale bar: 50 μm.

To determine the effect of Netrin-4 on VSMC adhesion, 96-well plates were coated with the recombinant human Netrin-4 protein (R&D), BSA (as a negative control) or Vitronectin (as a positive control). VSMC were allowed to adhere and spread for 4 hours and were then stained with crystal violet. The cells in wells coated with Vitronectin adhered (Figure 2A) and spread (Figure 2C), but cells on BSA did not adhere (Figure 2A). VSMC grown on Netrin-4-coated wells were adherent (Figure 2A) and showed a clear spreading of cells (Figure 2D). Measurement of dye absorbance confirmed these observations (Figure 2B). These results demonstrated that VSMC adhered to the Netrin-4 protein.

### VSMC express Netrin-4 and three of the six known Netrin receptors

**Figure 3:**
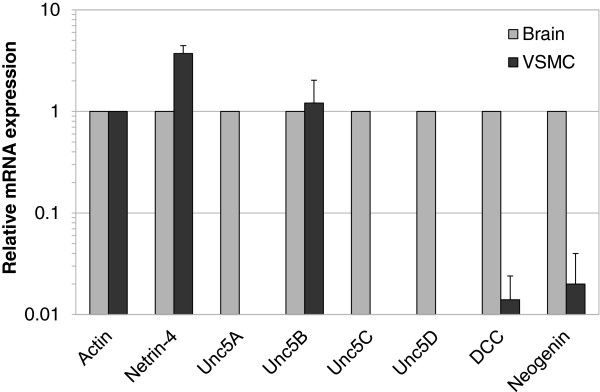
**Expression of Netrin-4 and Netrin receptors in VSMC.** Quantitative RT-PCR analysis of VSMC to assay Netrin-4 and Neogenin, DCC, and Unc5B receptor mRNA expression. Expression values were normalized to that of Actin and are reported relative to that in human fetal brain (normalized to 1).

We examined the expression of Netrin-4 and the six known Netrin receptors using VSMC from two different human donors and from Cambrex. Using quantitative real-time PCR, we detected mRNA encoding Netrin-4 and three Netrin receptors; DCC, Neogenin, and Unc5B, (Figure 3). Expression of Unc5A, Unc5C and Unc5D was not detected. Expression was measured as the amount of transcript relative to Actin and compared with transcript quantities present in human fetal brain (normalized to 1; Figure 3). We observed that DCC and Neogenin transcript levels were lower in VSMC compared with transcripts isolated from fetal brain tissue. In contrast, Netrin-4 transcript levels were higher in VSMC compared with transcripts isolated from fetal brain tissue (Figure 3). In contrast, in VSMC, the level of the Unc5B transcript was similar to that measured in the fetal brain (Figure 3; Additional file 2: Figure S2A).

**Figure 4:**
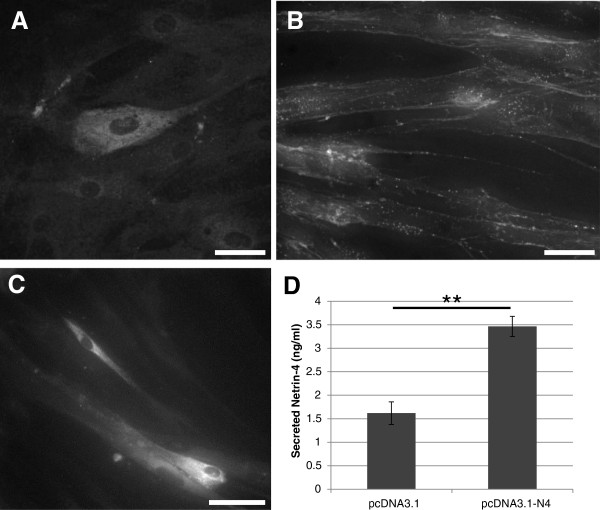
**Immunolabelling of endogenous and exogenous Netrin-4 in VSMC.** Cells were fixed and stained with an antibody against Netrin-4. **(A)** Immunolabelling of endogenous Netrin-4 within the cytoplasm of VSMC. **(B)** VSMC were incubated with exogenous Netrin-4 for 2 hours. Netrin-4 immunoreactivity is at the cell surface suggesting that the Netrin-4 is bound to the Netrin receptors. Scale bar: 100 μm. **(C & D)** Cells were transfected with empty vector (pcDN3.1) or with Netrin-4 expressing plasmid (pcDN3.1-N4). Two days after transfection, conditioned media were collected and cells were fixed. As observed in C, transfected cells express Netrin-4. Scale bar: 200 μm. **(D)** Detection of the protein in the conditioned media by ELISA showing that Netrin-4 is secreted by VSMC.

The presence of Netrin-4 in VSMC was further confirmed by immunocytochemistry. VSMC were fixed and stained with a specific antibody that recognizes the human Netrin-4 protein (R&D). Endogenous Netrin-4 was found distributed throughout the cytoplasm of VSMC (Figure 4A). To visualize a possible interaction between VSMC and exogenous Netrin-4, cells were incubated for 2 hours with the recombinant human protein (R&D). Netrin-4 immunostaining revealed Netrin-4 specifically localized on the cell surface and along cell processes (Figure 4B). Because Netrin-4 is a secreted protein, ELISA assays were performed on VSMC after transfection of empty vector or plasmid encoding Netrin-4 to determine whether VSMC secrete Netrin-4. Expression of Netrin-4 in transfected cells was visualized by immunocytochemistry (Figure 4C). Detection of the protein in VSMC conditioned media indicated that Netrin-4 was secreted from VSMC (Figure 4D).

### Netrin-4 induces VSMC adhesion via DCC, Neogenin and Unc5B receptors

**Figure 5:**
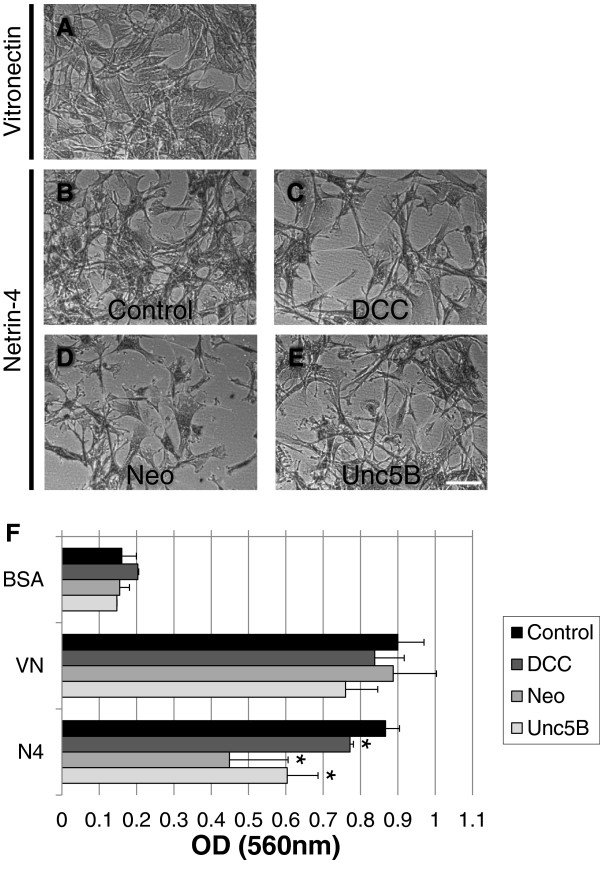
**DCC, Neogenin and Unc5B are required for Netrin-4-induced VSMC adhesion.** VSMC were transfected with a control non-specific siRNA (control; **A & B**) or a specific siRNA targeting the DCC **(C)**, Neogenin **(D)** or Unc5B **(E)** receptors. Contrast phase images indicate that transfected VSMC adhered to Vitronectin **(A)** and to Netrin-4 **(B-E)**. Scale bar: 150 μm. **(F)** Quantification of cell adhesion to the three substrates by measurement of absorbance under the four experimental conditions. Silencing of DCC, Neogenin or Unc5B significantly decreased VSMC adhesion to Netrin-4, implicating these three receptors in the process.

To determine which Netrin receptor expressed on VSMC mediated the observed response to Netrin-4, expression of the DCC, Neogenin and Unc5B Netrin receptors was silenced using siRNA in VSMC. Silencing was verified by RT-PCR 24 hours after transfection (Additional file 2: Figure S2B). Cell adhesion assays were performed two days after siRNA transfection as described in Figure 2. Transfected VSMC were allowed to adhere to BSA, Vitronectin or Netrin-4 coated culture wells. Transfected VSMC adhered to Vitronectin (Figure 5A) and Netrin-4 (Figures 5B, C, D, and E). Knocking-down DCC, Neogenin or Unc5B expression did not change the adhesion capacity of VSMC to Vitronectin (Figure 5F); however, silencing DCC, Neogenin or Unc5B receptors significantly reduced VSMC adhesion to Netrin-4 (Figure 5F), suggesting that all three receptors are involved in this process.

### Netrin-4 enhances VSMC recruitment in an *in vitro* model of endothelial cell-pericyte interaction

**Figure 6:**
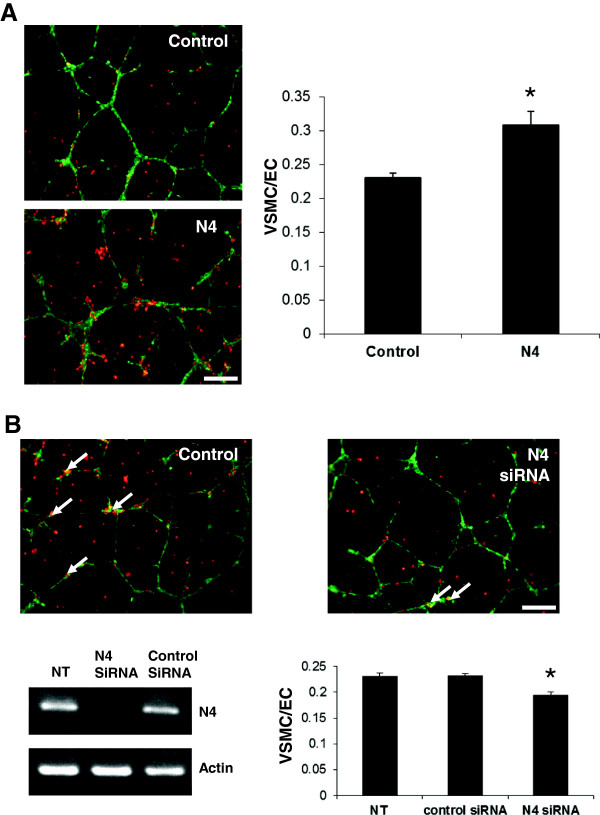
**Netrin-4 enhances VSMC recruitment on HUAEC *in vitro. *(A)** Co-culture of VSMC (stained in red) and HUAEC (stained in green) grown on Matrigel indicated that pericyte coverage was greater with VSMC pretreated with Netrin-4 (N4) compared with non-treated cells (Control). **(B)** Silencing of Netrin-4 expression in VSMC using siRNA significantly reduced HUAEC coverage by VSMC (arrows). RT-PCR analysis showed that N4 expression is down-regulated in VSMC transfected with Netrin-4 siRNA (N4siRNA), but not in non-transfected (NT) cells or in VSMC transfected with a control siRNA (Control siRNA). A Student’s *t* test was used to assess the statistical significance of differences between groups **P < 0.01, *P < 0.05.

Netrin-4 was shown to inhibit the ability of HUAEC to form vascular networks on Matrigel [17]. To investigate the effects of Netrin-4 on VSMC and their interaction with EC, we used a co-culture model of HUAEC and VSMC plated on Matrigel. HUAEC were plated on Matrigel and allowed to form vascular networks for 24 hours. VSMC were then added and were left in contact with HUAEC for five hours. Two experiments were performed using this HUAEC-VSMC co-culture assay. First, we pretreated VSMC with exogenous Netrin-4 for 24 h prior to co-culture with HUAEC. This pre-treatment significantly increased the percentage of HUAEC coverage by VSMC (Figure 6A). Second, expression of Netrin-4 in VSMC was silenced by siRNA to investigate the role of Netrin-4 in mural cell recruitment. RT-PCR analysis confirmed a specific down-regulation of Netrin-4 expression in VSMC transfected with a siRNA targeting Netrin-4 (Figure 6B). This silencing of Netrin-4 in VSMC significantly reduced their coverage of HUAEC (Figure 6B) implicating endogenous Netrin-4 expression by VSMC in adhesion of VSMC on EC.

### Overexpression of Netrin-4 by PC3 cells increases mural cell coverage in tumor vasculature

**Figure 7:**
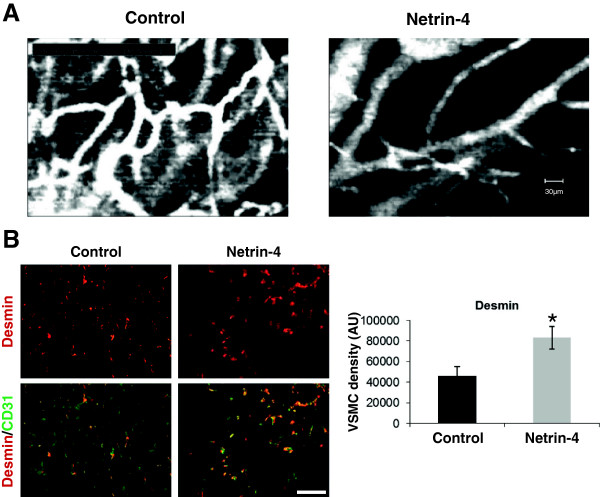
**Netrin-4 induces mural cell coverage of tumor vessels *in vivo. *(A)** Intravital microscopy of subcutaneous PC3 tumors (Control) and those overexpressing Netrin-4 (Netrin-4). The organization of blood vessels in Netrin-4 overexpressing tumors was better structured than in control tumors. Imaging probe field: 400 × 300 μm. **(B)** Desmin (red) and CD31 (green) immunostaining on tissue sections of four different tumors from Netrin-4 transfected (Netrin-4) and empty vector transfected PC3 (Control) groups. Fields were examined by immunofluorescence microscopy, data were analyzed by Image J software and results expressed as mean pixels per μm^2^ (20 fields per condition). A Student’s *t* test was used to assess the statistical significance of differences between groups *P < 0.05. Desmin staining was significantly greater in Netrin-4-overexpressing tumors (graph in B) and co-localized with CD31 immunostaining (merge: Desmin/CD31) indicating that Netrin-4 promotes VSMC coverage of tumor vessels.

We previously reported that Netrin-4 overexpression in PC3 cells delayed tumor uptake and growth. These Netrin-4 anti-tumor properties were attributed to direct effects on endothelial cells [17]. Here, we investigated the role of Netrin-4 in vessel maturation using the same *in vivo* model. Tumor blood vessel maturation was assessed in nude mice subcutaneously xenografted with human PC3 (prostate cancer) cells overexpressing Netrin-4. Intravital microscopy analysis of the morphology of intratumoral vasculature revealed that empty vector-transfected tumors were supplied by highly tortuous, disorganized vessels (Control, Figure 7A). In Netrin-4 transfected tumors, vessels displayed structure and hierarchical organization more similar to that of the microvascular network of healthy tissues (Netrin-4, Figure 7A). Immunohistochemical analysis of tumor sections with an antibody specific for desmin, a marker of perivascular cells, was used to evaluate the effect of Netrin-4 transfection on the tumor vasculature at the cellular level. The number of desmin-positive cells per slide was significantly higher in Netrin-4 transfected samples compared with controls (Figure 7B). Moreover, double immunostaining with the endothelial marker CD31 showed colocalization with desmin staining (Figure 7B) indicating that Netrin-4 enhanced the recruitment of perivascular cells on tumor EC.

Our *in vivo* findings suggested that Netrin-4 overexpression induced tumoral vessel maturation by recruiting more perivascular cells.

## Discussion

In the current study, we have investigated the role of Netrin-4 in VSMC during normal and pathological angiogenesis. *In vitro* analyses showed that Netrin-4 promotes VSMC migration and adhesion via DCC, Neogenin and Unc5B receptors. Silencing of Netrin-4 in VSMC resulted in a significant reduction in their association with EC tubes. Netrin-4 overexpression in a model of subcutaneous PC3 cell xenograft increased the recruitment of mural cells to tumor vessels.

### Netrin-4 promotes the recruitment of VSMC on EC-lined tubes formed in vitro

Angiogenesis is regulated by interactions between endothelial, mural and stromal cells with the extracellular matrix [32,33]. In addition, interactions between the cell types, EC and VSMC, are critical for the formation, maturation and maintenance of blood vessels (as reviewed in [1,2]); therefore, angiogenesis modulators should be assessed in both of these cell types. Netrin-4 is secreted by both EC and VSMC and so may act on different targets, involving each cell type separately, as well as acting on their interactions. We have previously demonstrated that Netrin-4 inhibits HUAEC proliferation and migration as well as their ability to form tubular structures on Matrigel [17]. Here, our findings clearly indicate that Netrin-4 influences *in vitro* VSMC recruitment to EC-lined tubes formed on Matrigel. Because silencing Netrin-4 expression in VSMC reduces their coverage on EC, our data indicate that endogenous Netrin-4 produced by VSMC is important for their recruitment. These data suggest that Netrin-4 secreted by VSMC may be required for their interaction with EC through Netrin receptors. Because VSMC express DCC, Neogenin and Unc5B receptors, Netrin-4 may also affect VSMC in a cell autonomous manner. Further experiments such as silencing receptor expression in the co-culture assay will be necessary to better understand Netrin-4 mechanism of action. Moreover, pre-treatment of VSMC with exogenous Netrin-4 increased EC coverage, suggesting that Netrin-4 produced by EC or stored in ECM may also play a role in VSMC recruitment.

Stratman *et al.* reported that EC are required to induce pericyte motility in a 3D collagen matrix and that EC production of PDGF and EGF regulates pericyte coverage [34,35]. Here, we show that Netrin-4 enhances VSMC migration, adhesion and association to EC networks on Matrigel. Both VSMC and EC produce Netrin-4 and express Netrin-4 receptors, which have been shown to mediate VSMC adhesion and migration (Additional file 2: Figure S2C). Because silencing all three Netrin VSMC receptors does not completely inhibit adhesion to Netrin-4 (data not shown), other receptors, such as integrins [36-39], may also be involved. Specific intercellular signals are required to mediate the EC-pericyte interaction [40]. Thus, the Netrin-4 pathway (Netrin-4 and its receptors present on EC and VSMC) may play an important role in this endothelial-pericyte communication.

### Netrin-4 may recruit mural cells as a component of the vascular BM

Interaction of EC and perivascular cells with the basement membrane (BM) also plays an important role during the angiogenic processes [22]. EC and pericytes both contribute to basement membrane composition, deposition and assembly. ECM components influence the behavior of EC and perivascular cells. BM components appear to be as guiding cues for pericytes that extend cellular processes to contact EC [35]. Genetic ablation of Neuron-glial proteoglycan 2 (NG-2), a cell surface component of pericyte, impairs the interaction between EC and pericytes, which leads to reduced pericyte attachment and assembly of the vascular basal lamina. Further analysis of neovessels in NG2 null mice revealed changes in components of the basal lamina including loss of collagen VI and reduced collagen IV deposition [41]. Thickening of the basement membrane and selective loss of pericytes are early events of human diabetic retinopathy [28]. Thus pericyte adhesion and formation of the perivascular matrix seem to be linked and play essential roles for vascular stability.

In vessels, the BM is mainly composed of four types of molecules including laminin, collagen, perlecan and nidogen [42]. Netrin-4 is structurally similar to laminin beta chains and is often deposited in basement membranes. Netrin-4 is a component of the vascular basal lamina [23] and has been reported to interact with laminins [24]. Laminins are a major family of ECM molecules and play an important role in basement membrane assembly in angiogenesis [42]. Binding of Netrin-4 to laminin γ1 short arms inhibits laminin self assembly [24]; Netrin-4 may act through interaction with basement membrane components such as laminin, which can then influence signaling to EC and mural cells.

### Netrin-4 promotes mural cell recruitment on tumor blood vessels

Most studies have focused on the role of EC in tumor angiogenesis; however, pericytes are commonly involved in tumor angiogenesis. As such, there is currently interest in their potential as targets for cancer therapies [3,43]. There are only a few reports on the expression of Netrin-4 in human cancer [44-46] although Netrin-4 has been recently identified as a gene up-regulated in 37.5% of breast cancers and its expression is correlated with longer overall survival [45]. We reported previously that Netrin-4 overexpression in pancreatic tumor cells (PC3) [17] and in colon cancer cells (LS174) [18] delayed tumor angiogenesis in two mouse models of subcutaneous xenograft. These properties were first attributed to direct effects on endothelial cells. Here, we addressed the role of Netrin-4 on mural cells using the same PC3 model. Double Desmin/CD31 immunostaining of tumor sections shows that Netrin-4 overexpression in PC3 cancer cells increases coverage of tumor vasculature. However, further characterization of these desmin positive cells would be necessary to confirm the identity of these perivascular cells. Furthermore, intravital microscopy analysis of tumors shows that Netrin-4 overexpression correlated with vessel normalization.

Taken together, our *in vivo* findings suggest that Netrin-4 overexpression induces tumor vessel maturation by recruiting more perivascular cells. In this model, the main source of Netrin-4 is provided by the transfected PC3 tumor cells, which secrete the protein. Thus, our results suggest that it is the secreted Netrin-4 that promotes the recruitment of mural cells on the nascent vasculature, contributing to blood vessel maturation and stabilization. This is also supported by our *in vitro* observation that Netrin-4 enhances the EC coverage by VSMC*.* Overexpression of Netrin-4 may also mediate tumor vascular normalization through its actions on the vascular basement membrane and inhibitory effects on EC. As observed in a recent model of corneal neovascularization, Netrin-4 may also induce tumor blood vessels normalization through down-regulation of VEGF [47]. Finally, overexpression of Netrin-4 could restore the balance between pro-and anti-angiogenic signaling.

The *in vivo* analysis also suggests that maturation of the tumoral vasculature induced by Netrin-4 may be responsible for the reduced tumor growth *in vivo*. Overexpression of PDGF-BB in pancreatic and colorectal cancer cells leads to an increase in pericyte recruitment to the tumor vasculature, which in turn slows down tumor growth [48]. Pericyte recruitment is essential for the development of the tumor vasculature, and is dependent on various angiogenic factors. There are two possible strategies for anti-tumoral therapies targeting angiogenesis. Increasing the number of pericytes may stabilize and normalize blood vessels, thereby restricting vessel sprouting and tumor growth. Alternatively, limiting pericyte coverage may also lead to destabilization and collapse of the vasculature. Both strategies induce tumor regression [3,33]. Promoting pericyte coverage of tumor vessels may not only prevent vessel sprouting and tumor uptake but may also allow more effective intratumoral delivery of chemotherapy. Therefore, normalization of tumor vessels may be valuable for anti-angiogenic therapy [49]. A recent study supports this view: a blockade of the TGF-beta signaling pathway increased pericyte recruitment and incorporation into tumor vasculature. Normalization of tumor blood vessels reduced cancer progression in terms of tumor growth and metastasis, suggesting improved drug delivery and efficacy [50].

In addition to tumor pericyte coverage, the degree of pericyte involvement may influence the dynamics of tumor growth. Abnormal pericyte integration may be responsible for vessel defects that contribute to metastasis. Defects in pericyte coverage of the tumor vasculature are associated with an increase in the number of metastases in several mouse models of cancer [3]. Netrin-4 overexpression decreases colorectal cancer progression in terms of liver metastasis number and size [51]. Finally, tumor blood vessels normalization may represent a mechanism that interferes with tumor growth. Thalidomide is an anti-tumor and anti-angiogenic factor whose properties were attributed to direct effects on endothelial cells. Interestingly, a recent study demonstrated that thalidomide also promotes vessel maturation by enhancing pericyte coverage, suggesting that therapeutic effect of this compound might be the result of inhibition of endothelial cells as well as of direct stimulation of mural cells recruitment [52].

In conclusion, Netrin-4 appears to stimulate mural cell recruitment (this study) and limit EC proliferation [17]. We propose that Netrin-4 acts as an anti-angiogenic factor through the regulation of both endothelial and perivascular cells.

## Endnotes

### Non standard abbreviations used

VSMC: vascular smooth muscle cells; EC: endothelial cells; HUAEC: Human Umbilical Arterial Endothelial Cells; N4: Netrin-4; ECM: extracellular matrix; BM: basement membrane.

## Competing interest

The authors have declared no conflict of interest.

## Authors’ contributions

Designed Research: LL, OC, JP. Performed Research: EL, IB, SC, MR, CLD, NM, TMR, GA, CV, LL, JP. Analyzed data: EL, SC, NM, LL, JP. Wrote the paper: SC, LL. All authors read and approved the final manuscript.

## Authors’ information

This manuscript is dedicated to Dr Jean Plouët.

## Supplementary Material

Additional file 1: Figure S1 Netrin-4 does not stimulate mural cell proliferation: MTT cell proliferation assay of Vascular Smooth Muscle Cells **(A)** and Porcine Retinal Pericyte Cells **(B)** incubated for 24 hours with or without Netrin-4. Serum free condition was used as a negative control and cells incubated in their complete growth medium (Control) was used as a positive control. Netrin-4 (N4) was added to the complete medium at the indicated final concentration. Addition of Netrin-4 did not stimulate cell proliferation. No significant differences were observed between the control and the Netrin-4 conditions thus indicating that Netrin-4 does not influence cell proliferation. Click here for file

Additional file 2: Figure S2 **(A)** RT-PCR analysis of Netrin-4 and Netrin receptors in human VSMC and fetal brain. VSMC Netrin-4 and UNC5B transcript levels are similar compared with those of measured in the fetal brain; however, expression of Neogenin and DCC receptors was lower in VSMC compared with the brain. **(B)** RT-PCR analysis of Netrin receptors after siRNA transfection: Cells were transfected with either Control (Ctrl), DCC, Neogenin (Neo) or UNC5B siRNAs. Expression of Netrin receptors and Actin was measured via RT-PCR analysis in the four different conditions. Actin was expressed in all samples. Expression of Unc5B was not detected in cells treated with siRNAs targeting UNC5B. Neogenin and DCC expression was down-regulated in the cells transfected with the corresponding siRNA. **(C)** Effect of Netrin receptors invalidation on VSMC migration: Time course migration of non-transfected cells (NT) and cells transfected with the control siRNA (Control) or with a SiRNA targeting a netrin receptor (DCC, Neogenin and Unc5B). After 30 hours post-transfection, cells were allowed to migrate for 18 h in the presence of 50 ng/ml of Netrin-4. Compared with the control, N4-induced migration of VSMC was decreased when the DCC or Neogenin receptor expression was decreased, thus implicating these receptors in the process. Click here for file
