# Supplementary figures and images for: Netrin-4 promotes mural cell adhesion and recruitment to endothelial cells
Source: Vasc Cell. 2014 Jan 28;6:1. doi: 10.1186/2045-824X-6-1 (PMC3909532; doi:10.1186/2045-824X-6-1)

**A**

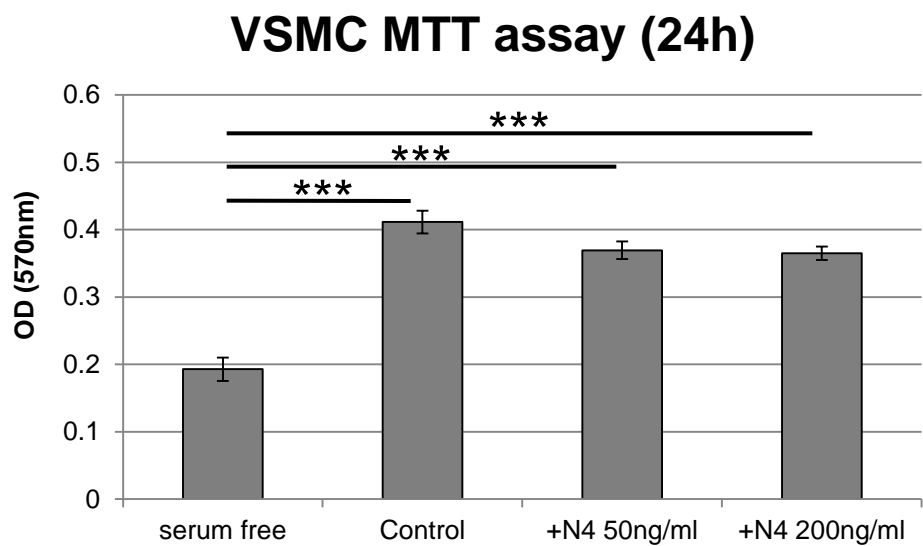

**B**

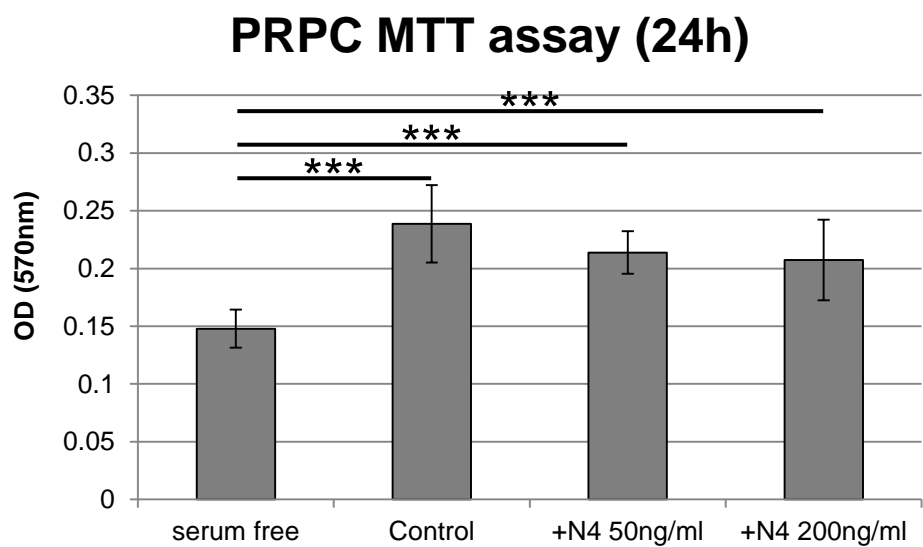

Supplement: Additional file 1: Figure S1 — Netrin-4 does not stimulate mural cell proliferation: MTT cell proliferation assay of Vascular Smooth Muscle Cells (A) and Porcine Retinal Pericyte Cells (B) incubated for 24 hours with or without Netrin-4. Serum free condition was used as a negative control and cells incubated in their complete growth medium (Control) was used as a positive control. Netrin-4 (N4) was added to the complete medium at the indicated final concentration. Addition of Netrin-4 did not stimulate cell proliferation. No significant differences were observed between the control and the Netrin-4 conditions thus indicating that Netrin-4 does not influence cell proliferation. [file 2045-824X-6-1-S1.pdf]

Additional file: Figure S2

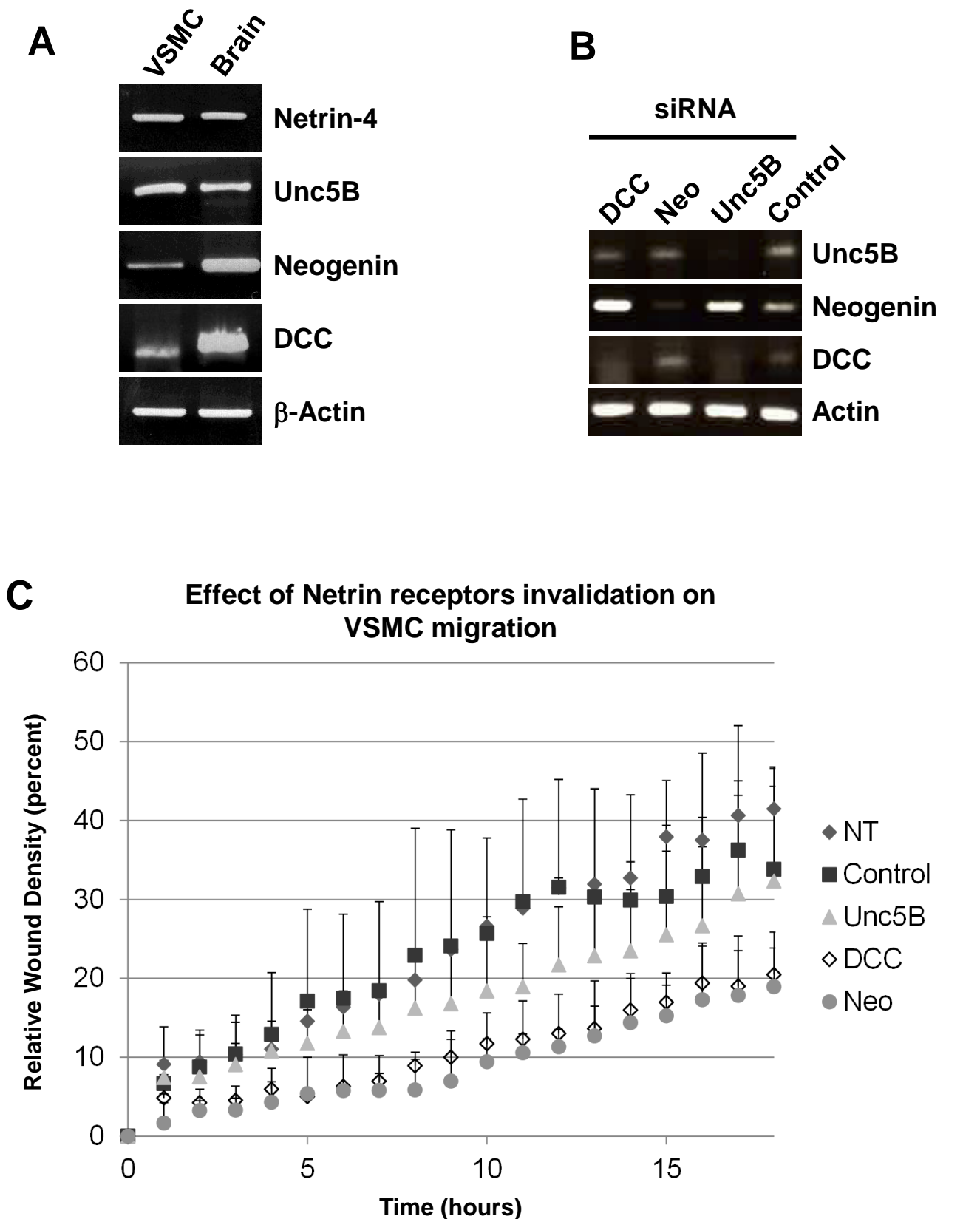

Supplement: Additional file 2: Figure S2 — (A) RT-PCR analysis of Netrin-4 and Netrin receptors in human VSMC and fetal brain. VSMC Netrin-4 and UNC5B transcript levels are similar compared with those of measured in the fetal brain; however, expression of Neogenin and DCC receptors was lower in VSMC compared with the brain. (B) RT-PCR analysis of Netrin receptors after siRNA transfection: Cells were transfected with either Control (Ctrl), DCC, Neogenin (Neo) or UNC5B siRNAs. Expression of Netrin receptors and Actin was measured via RT-PCR analysis in the four different conditions. Actin was expressed in all samples. Expression of Unc5B was not detected in cells treated with siRNAs targeting UNC5B. Neogenin and DCC expression was down-regulated in the cells transfected with the corresponding siRNA. (C) Effect of Netrin receptors invalidation on VSMC migration: Time course migration of non-transfected cells (NT) and cells transfected with the control siRNA (Control) or with a SiRNA targeting a netrin receptor (DCC, Neogenin and Unc5B). After 30 hours post-transfection, cells were allowed to migrate for 18 h in the presence of 50 ng/ml of Netrin-4. Compared with the control, N4-induced migration of VSMC was decreased when the DCC or Neogenin receptor expression was decreased, thus implicating these receptors in the process. [file 2045-824X-6-1-S2.pdf]
